# Attitudes, knowledge, and perceptions among women toward uterus transplantation and donation in the United Kingdom

**DOI:** 10.3389/fmed.2023.1223228

**Published:** 2023-08-16

**Authors:** Saaliha Vali, Benjamin P. Jones, Sairah Sheikh, Srdjan Saso, Isabel Quiroga, J. Richard Smith

**Affiliations:** ^1^West London Gynaecological Cancer Centre, Hammersmith Hospital, Imperial College NHS Trust, London, United Kingdom; ^2^Department of Metabolism, Digestion and Reproduction, Imperial College London, London, United Kingdom; ^3^Cutrale Perioperative and Ageing Group, Sir Michael Uren Hub, Imperial College London, London, United Kingdom; ^4^Queen Mary University of London, London, United Kingdom; ^5^The Oxford Transplant Centre, The Churchill Hospital, Oxford University Hospitals NHS Trust, Oxford, United Kingdom

**Keywords:** gynecology, infertility, transplantation, organ donation, MRKH

## Abstract

**Objective:**

To assess the motivations and perceptions of the general public in the United Kingdom toward donating their uterus for Uterus Transplantation after death (UTx).

**Design:**

A cross sectional study.

**Setting:**

A 32-item electronic questionnaire.

**Population:**

One hundred fifty nine females over the age of 16 living in the United Kingdom, consented and took part in the study.

**Main outcome measures:**

The motivations and perceptions toward UTx among the general public including the willingness to donate and barriers preventing donation.

**Results:**

One hundred fifty nine women completed the questionnaire. The majority had never heard of UTx (*n* = 107, 71%) and most were not aware the uterus could be donated after death (*n* = 130, 92%). 43% of the cohort were willing to donate their uterus after death (*n* = 57). 8% stated they wished to donate their organs but not their uterus (*n* = 10). 30% of women (*n* = 42) believed the child born following UTx would have genetic links to the donor. Over half of the respondents (*n* = 65, 51%) strongly agreed or agreed they would feel joy in the knowledge that donation would lead to bringing a new life into the world. A quarter of respondents strongly agreed or agreed (*n* = 45, 25%) that the use of their uterus by another woman would feel like an extension of life.

**Conclusion:**

The findings indicate a favorable opinion toward UTx and a positive attitude toward donation of the uterus after death among the general public in the United Kingdom. The findings also highlight the need for education around UTx now this therapeutic option is available.

## Introduction

Uterus Transplantation (UTx) is fast becoming an established treatment option for women with Absolute Uterine Factor Infertility (AUFI) with over cases performed globally and 40 live births as a result (1). The majority of cases to date have been undertaken in women with Mayer Rokitansky Kuster Hauser syndrome (MRKH)- a congenital disorder resulting in the absence of the uterus. The remainder of UTx cases have been performed in women who have previously undergone a hysterectomy or who have severe Ashermans syndrome (2). The surgical procedure is evolving and lessons learned have been shared with the transplant community (3, 4). The technical success (deemed as a viable graft on postoperative day 30) for both living and deceased donor UTx recipients in the United States is reported to be 76% (5). Among the first 45 cases performed globally, the graft survival rate has been similar at 71.4% with the remaining 28.6% of grafts requiring a hysterectomy (6).

There has been an overall graft failure rate of 23% post uterus transplantation, in most cases secondary to thrombosis within the uterine vessels. Robotic retrieval of the uterus in a living donor has shown a lower graft failure rate of 12% (1) Immunosuppression regimes in uterus transplantation are largely based on experience in solid organ transplants. The regime used in our deceased donor study, the Investigational Study Into the Transplantation of the Uterus (INSITU) in the United Kingdom is maintenance immunosuppression with tacrolimus and Mycophenolate Mofetil (MMF) for the first 3 months post transplantation after which the MMF is switched to azathioprine due to its associated increased risks of congenital malformation in early pregnancy. Where a CMV seronegative recipient has received a uterus from a CMV seropositive donor (D+/R-), the risk of CMV seroconversion needs to be lowered with prophylactic medication such as Valganciclovir. Primary CMV infection poses a risk not only to the recipient but also to the fetus if she is pregnant. Primary CMV infections during pregnancy carry a 30–40% risk of transmission to the fetus (7). Thus, most UTx programs avoid recipient-donor pairs where there is a CMV status mismatch.

Uterus donation after brainstem death (DBD) refers to the retrieval of the uterus during a multi-organ retrieval, from a donor who has been pronounced brain stem dead. The uterus has been successfully retrieved at the beginning of a multi-organ retrieval, and after the retrieval of the other solid organs, with no negative impact upon other organs retrieved or the retrieval process (8–13). The first livebirth following DBD UTx was achieved in Brazil in 2017 demonstrating the feasibility of UTx using deceased donors (14). UTx teams worldwide have continued to pursue this route alongside living donor UTx, though logistical difficulties make it more complicated to create a sustainable program. Ethically, UTx involving deceased donors is more favorable to living donors given the elimination of surgical risk to the donor. However, one of the limiting factors for the success of sustainability of clinical UTx programs is the limited supply of uteri from DBD donors.

The age of the uterus donor has shown to have an impact on the outcome, largely due to the risk of graft-vessel thrombosis (15). Thus, younger donors are preferred to increase the likelihood of a better quality graft.

AUFI has been estimated to affect one in 500 women of reproductive age, which equates to 30,000 women in the United Kingdom alone, although, only a minority of these would be eligible and have the desire to undergo UTx (14). The uterus is not currently part of the opt out system for organ donation which was introduced in the United Kingdom in 2020 and therefore requires separate consent from family members after the donor’s death (16). While 785 DBD retrievals were undertaken between April 2021–2022, only 43% were undertaken in females and only 35% were between the ages of 18–50 (17). Therefore, we anticipate that between 120 and 130 donors will be broadly eligible for uterine retrieval each year. On the basis of current selection criteria, including necessity for being parous, having an uncomplicated obstetric history, and lack of significant medical problems, this number will decrease significantly further (17, 18). Following donor family consent and authorization, and subsequent pre-operative investigations, it is expected that DBD donors who fully meet the criteria for uterine retrieval will be less than 50 women per year in the United Kingdom (19).

Given the limited availability of deceased donors, the attitudes toward donation in the general public need to be explored. Research has shown factors that influence donor decisions include medical mistrust and concerns around bodily integrity, issues which remain paramount among ethnic groups where organ donation remains a challenge. Medical mistrust issues mainly consist of concerns around the organ being used for research purposes and fears that the brain death diagnosis would be accelerated in registered donors (20, 21). As these are not specifically cultural or religious barriers to donation, but rather misconceptions, it may be possible to alleviate these through targeted education campaigns.

The success of a clinical UTx program is dependent on women expressing a wish to donate their uterus and the donor families consenting to uterine donation. Now that the Investigational Study into the Transplantation of the Uterus (INSITU) study is live in the United Kingdom, and with the intention of creating a sustainable UTx program using both living and deceased donors in the United Kingdom, it is essential to gage the attitudes of the general public toward UTx.

### Study objective

To assess the motivations, perceptions and number of women in the United Kingdom who would be willing to donate their uterus for UTx.

## Methods

The study was advertised through social media platforms including Instagram, Facebook, Twitter and local university networks. The criteria for inclusion were female, age over 16 years and residency in the United Kingdom. The study information sheet (Appendix 1) was attached to the advertisement which included a brief overview of AUFI, UTx and alternative options for motherhood. An electronic link to the online consent form was included which required completion in its entirety prior to proceeding with the questionnaire. Participants were recruited over a four-month period.

The questionnaire consisted of 32 items (Appendix 2) and was constructed on the SurveyMonkey platform. The survey items recorded demographic information, knowledge and awareness on UTx, perceptions toward UTx and organ donation in general. All questions were closed- ended with pre populated options mostly being yes/no and the strongly agree to strongly disagree five-point Likert scale for questions relating to perceptions. The study followed the American Association for Public Opinion Research (AAPOR) principles on reporting survey- based research. Ethical approval to undertake the study was received from Imperial College London, United Kingdom (reference 21IC6620). No personally identifiable data was collected thus all responses remained anonymous.

### Statistical analysis

Data was analyzed using descriptive statistics. The Likert responses were quantified using the weighted ranking system to ascertain the strongest sentiment among the group. Each sentiment level on the scale was assigned a value, from 1 assigned to “strongly disagree” ranging to 5 assigned to “strongly agree”. SPSS software, version 26 (SPSS Inc) was used for statistical analysis.

## Results

**Figure 1:**
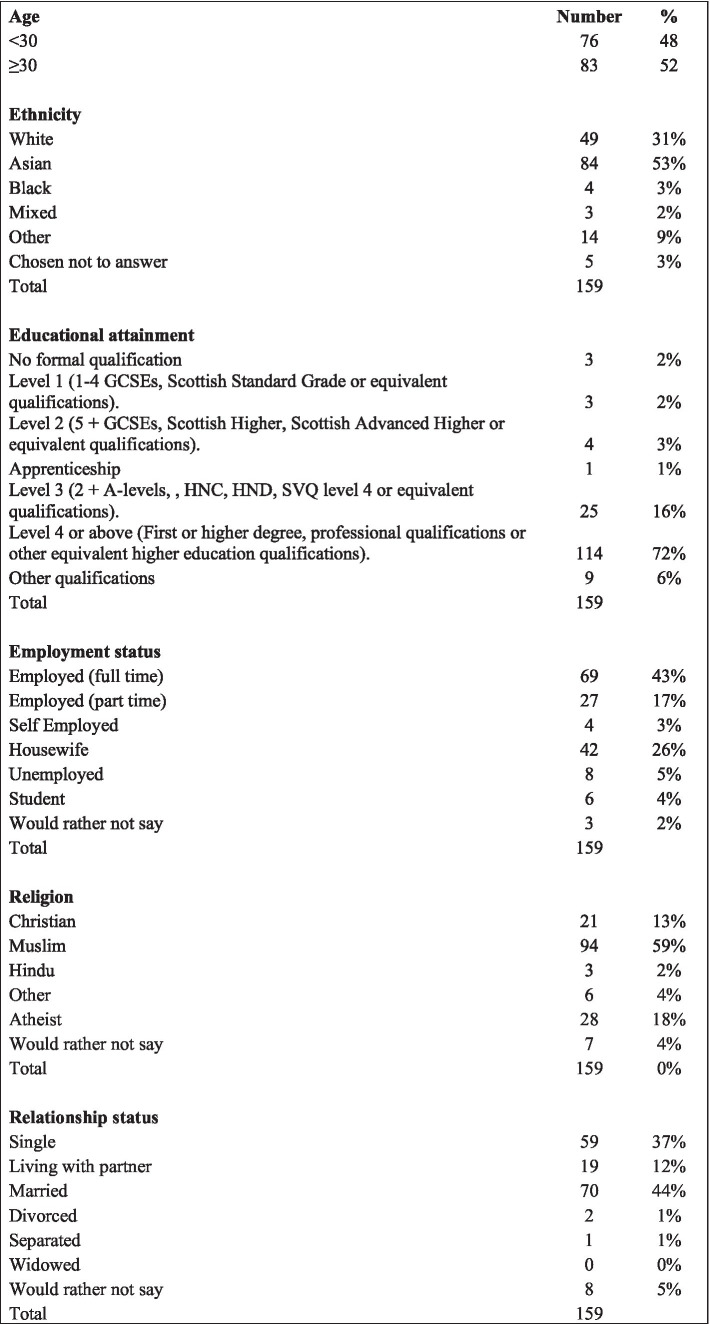
Demographic details of participants.

A total of 171 participants consented to participate in the study and of these, 159 completed the questionnaire, resulting in a response rate of 93%. Participant demographics are summarized in Figure 1. The majority of participants were 30 years and above (*n* = 83, 52%). Most participants (*n* = 114, 72%) had attained an educational level of level 4 and above and 44% reported being in full time employment. The largest represented ethnic group among participants was Asian (*n* = 84, 53%), followed by White (*n* = 49, 31%). The most prevalent faith group was Muslim (*n* = 94, 59%) followed by Atheist (*n* = 28, 18%) and Christian (*n* = 21, 13%). Similar proportions of participants were married (*n* = 70, 44%) and single (n = 59, 37%).

**Figure 2:**
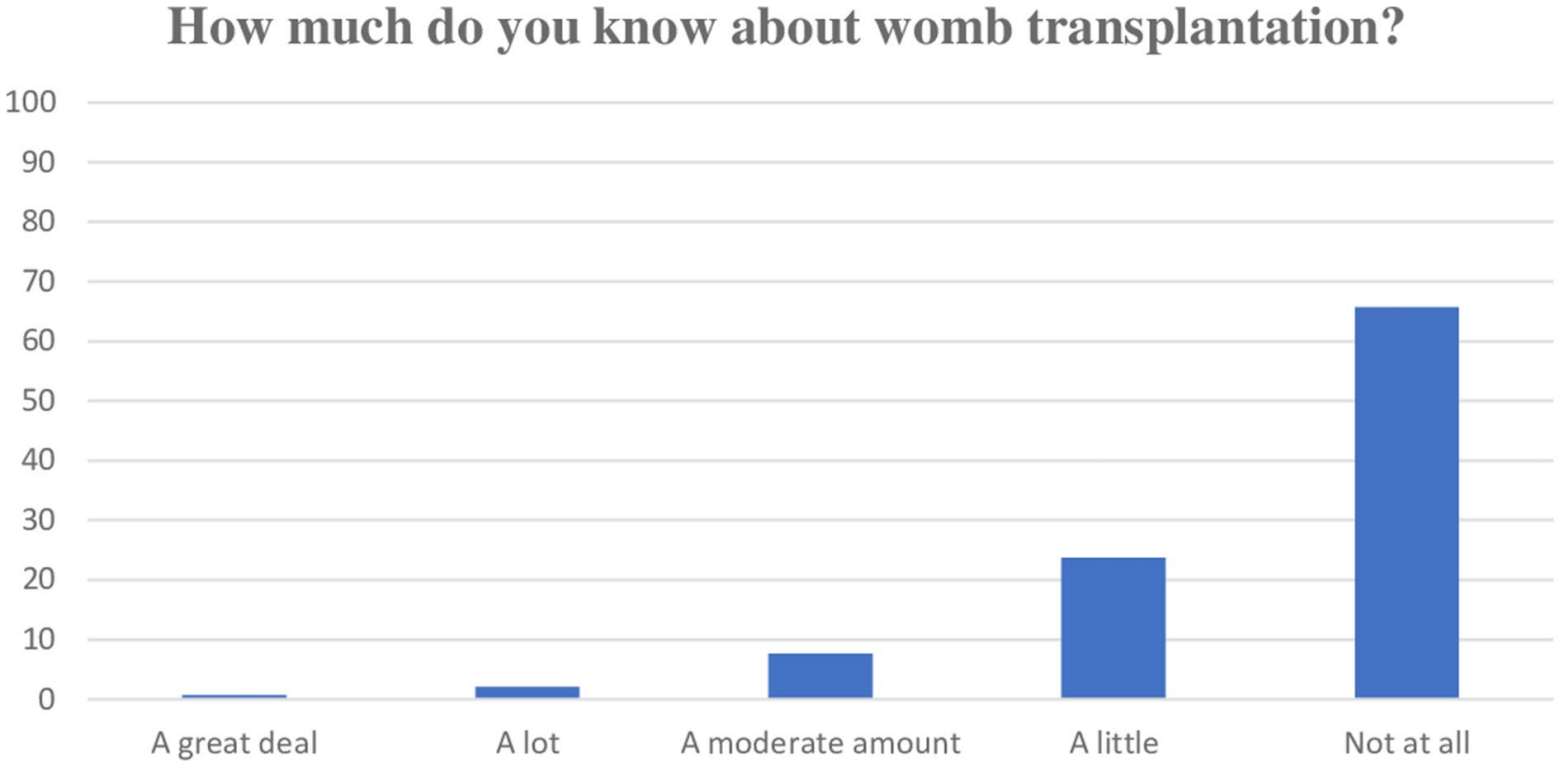
Knowledge of UTx.

**Figure 3:**
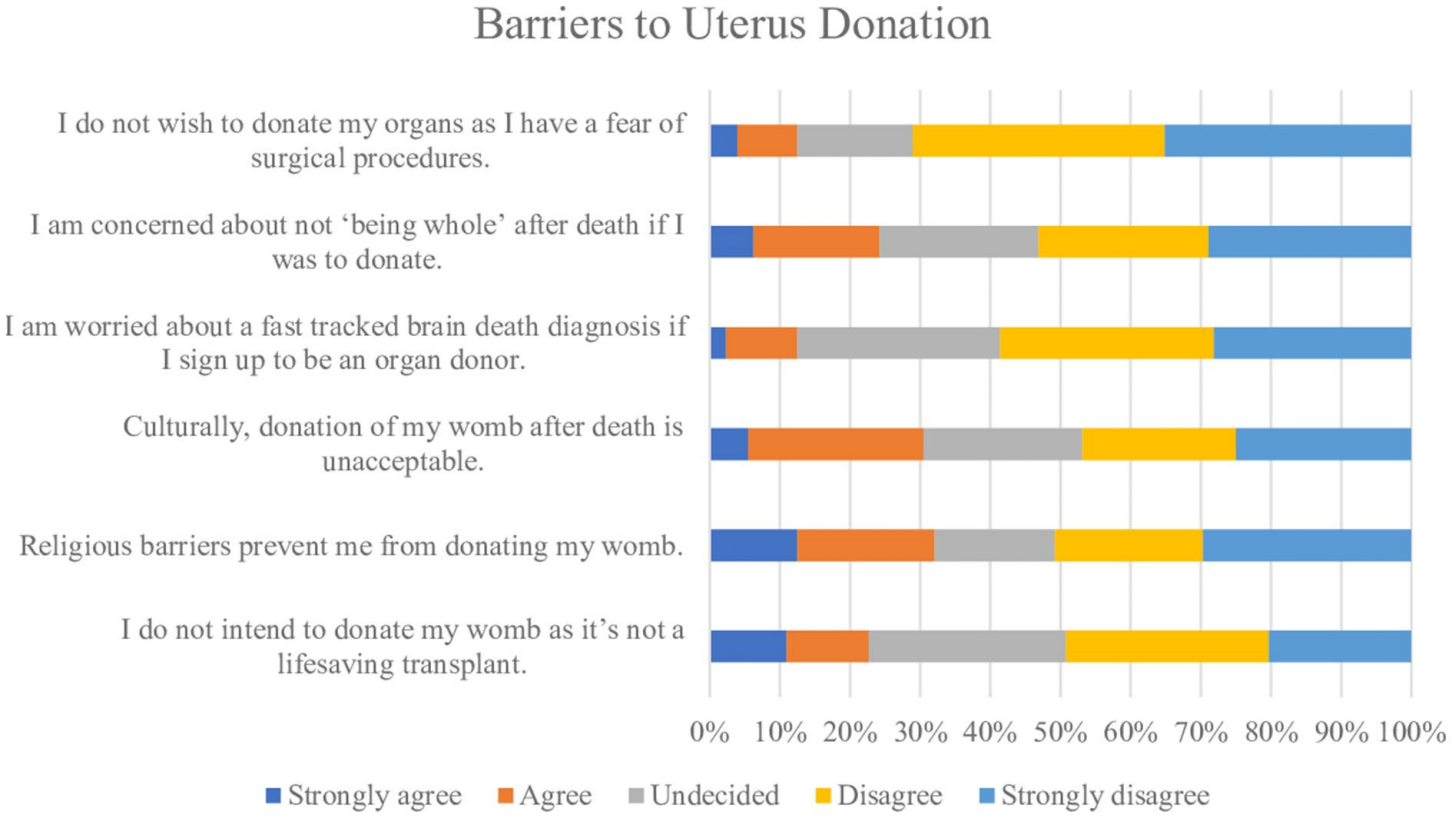
Barriers to donation of the uterus.

Knowledge and barriers toward organ donation in general were explored (Figures 2, 3) One hundred and twenty (78%) participants were aware of the opt-out system for organ donation introduced in May 2020. Among them, 48% (*n* = 58) were below age 30 and 52% (*n* = 62) were ≥ 30 years. A quarter (25%; *n* = 38) of participants reported having opted out and a further 28% (*n* = 43) reported intending to opt out. Among this group, 59% (*n* = 48) were below age 30 and 41% (*n* = 32) were ≥ 30 years. Only 11% (*n* = 14) of the total cohort reported they do not agree with organ donation in general and 13% (*n* = 16) reported they did not wish to donate their organs due to a fear of surgical procedures. Most participants (*n* = 75, 58%) strongly disagreed or disagreed on being worried about a fast-tracked brain death diagnosis if they were on the organ donor register. Similarly, over half (*n* = 68, 53%) strongly disagreed or disagreed on concerns around ‘being whole’ if they were to donate their organs. Eighty participants (63%) strongly disagreed or disagreed that their decisions around donation of their organs were affected by their family disagreeing to the process.

The majority of participants did not know of anybody who had received a donated organ (*n* = 106, 69%) and among this group there was a similar distribution among the faith groups Christian (*n* = 18, 90%), Muslim (*n* = 63, 79%), Hindu (*n* = 4, 100%) and Atheist (*n* = 23, 85%).

Similarly, the majority of participants (*n* = 127, 83%), responded they did not know anybody who had donated an organ and among the faith groups, the distribution was also similar [Christian (*n* = 2, 15%), Muslim (*n* = 5, 28%), Hindu (0%) and Atheist (*n* = 3, 13%)].

Over three quarters of respondents (77%) were unaware the uterus was not included on the opt out organ donation register. Among them, 31% (*n* = 11) were below age 30 and 69% (*n* = 24) were ≥ 30 years. However, over half of respondents (*n* = 66, 51%) strongly agreed or agreed to believing organ donation including the uterus, was a means of giving back to society.

One hundred and seven (71%) participants had never heard of UTx and of those who had, 37% (*n* = 21) had heard of UTx through the media and a further 37% (*n* = 21) had heard of the concept through a friend. Most of the participants (*n* = 130, 92%) were not aware the uterus could be donated after death.

Most participants (*n* = 128, 90%) claimed to know a little or nothing at all on UTx (Figure 2). Thirty percent (*n* = 42) believed the child born from a transplanted uterus would have genetic links to the deceased donor uterus. Within this group, 52% (*n* = 22) were below age 30 and 48% (*n* = 20) were ≥ 30 years.

**Figure 4:**
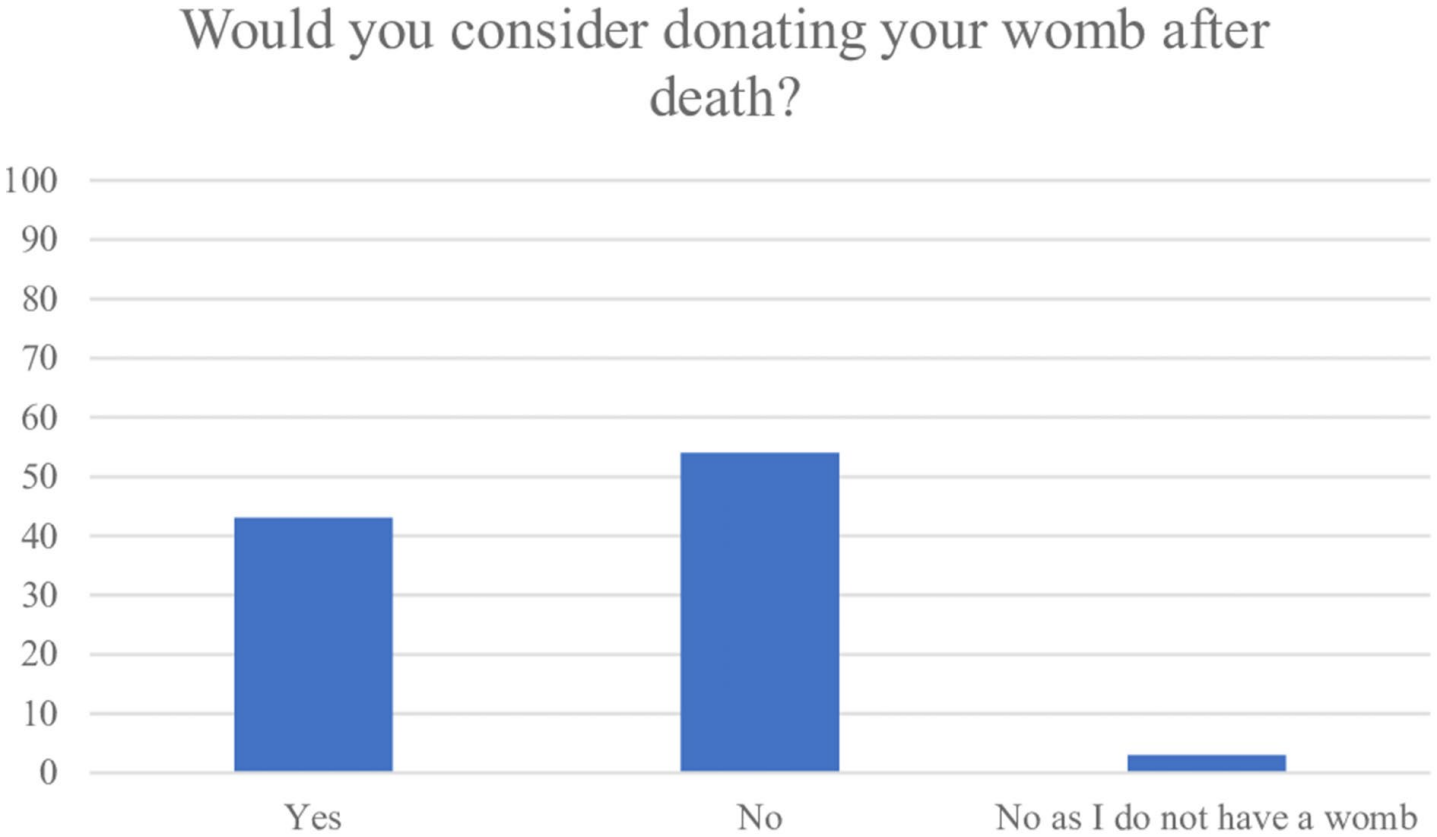
Number of women willing to donate their uterus after death.

43% (*n* = 62) of participants answered they would consider donating their uterus after death (Figure 4). There were 3% (*n* = 4) who reported they did not have a uterus. Subgroup analyses revealed variation among the faith groups on the intention to donate the uterus, with the most likely faith group being Muslim (*n* = 60, 74%) followed by Christian (*n* = 13, 65%) and Hindu (*n* = 2, 50%). A large proportion of Atheist’s (*n* = 24, 89%) responded they would consider donating their uterus after death. More than half of the women strongly disagreed or disagreed that religious barriers (*n* = 65, 51%) and cultural barriers (*n* = 60, 47%) would prevent them from donating their uterus. However, among the group who were considering to donate their uterus, cultural differences were observed: 83% (*n* = 38) of White, 23% (*n* = 17) Asian, 33% (*n* = 1) Black, 33% (*n* = 1) Mixed and 38% (*n* = 5) of participants identifying as “Other” ethnicity responded in the positive. There was little variation among the age groups on willingness to donate their uterus (less than 30 years n = 28, 45%, ≥30 years *n* = 34, 55%) and similarly among those not willing to donate their uterus after death (less than 30 years *n* = 39, 51% ≥30 years *n* = 38, 49%).

Among the group considering to donate their uterus, there were 88% (*n* = 46) of participants, who strongly agreed or agreed on planning to donate other organs and seeing the uterus as no different.

There were 54% of women who answered they would not consider donating their uterus after death (*n* = 77) and of these 70% (*n* = 19) either strongly agreed, agreed or were undecided when asked if they disagree with organ donation in general. Additionally, when asked how they feel about a vaginal examination to assess suitability of the womb after death, 42% (*n* = 14) remained undecided and 30% (*n* = 10) reported they strongly disagree or disagree.

Only 8% (*n* = 10) of all participants stated they wished to donate their organs but not their uterus.

Most respondents who would consider donating their uterus strongly agreed or agreed (*n* = 48, 98%) they would feel joy in the knowledge that donation of their uterus would lead to bringing a new life into the world and all (*n* = 28, 100%) strongly agreed or agreed that the use of their uterus by another woman would feel like an extension of life. Similarly, within the group considering to donate their uterus, 100% (*n* = 52) strongly agreed or agreed all organs including the womb are precious resources which are best put to use after death.

More than half of all respondents (*n* = 89, 58%) did not have children but of these, 86% (*n* = 76) intended to have children in the future. Most respondents (*n* = 80, 91%) had not experienced infertility themselves. Interestingly, 36% (*n* = 26) of women who intended to have children in the future responded they would consider donating their uterus after death, a contrast to the 58% (*n* = 7) of women who do not intend to have children and willing to donate their uterus after death. Additionally, only 38% (*n* = 3) of women who answered they have personally experienced infertility also considered donating their uterus after death. A higher number of women who answered knowing somebody personally who is experiencing infertility (*n* = 104, 71%) also answered they would consider donating their uterus (*n* = 49, 47%).

**Figure 5:**
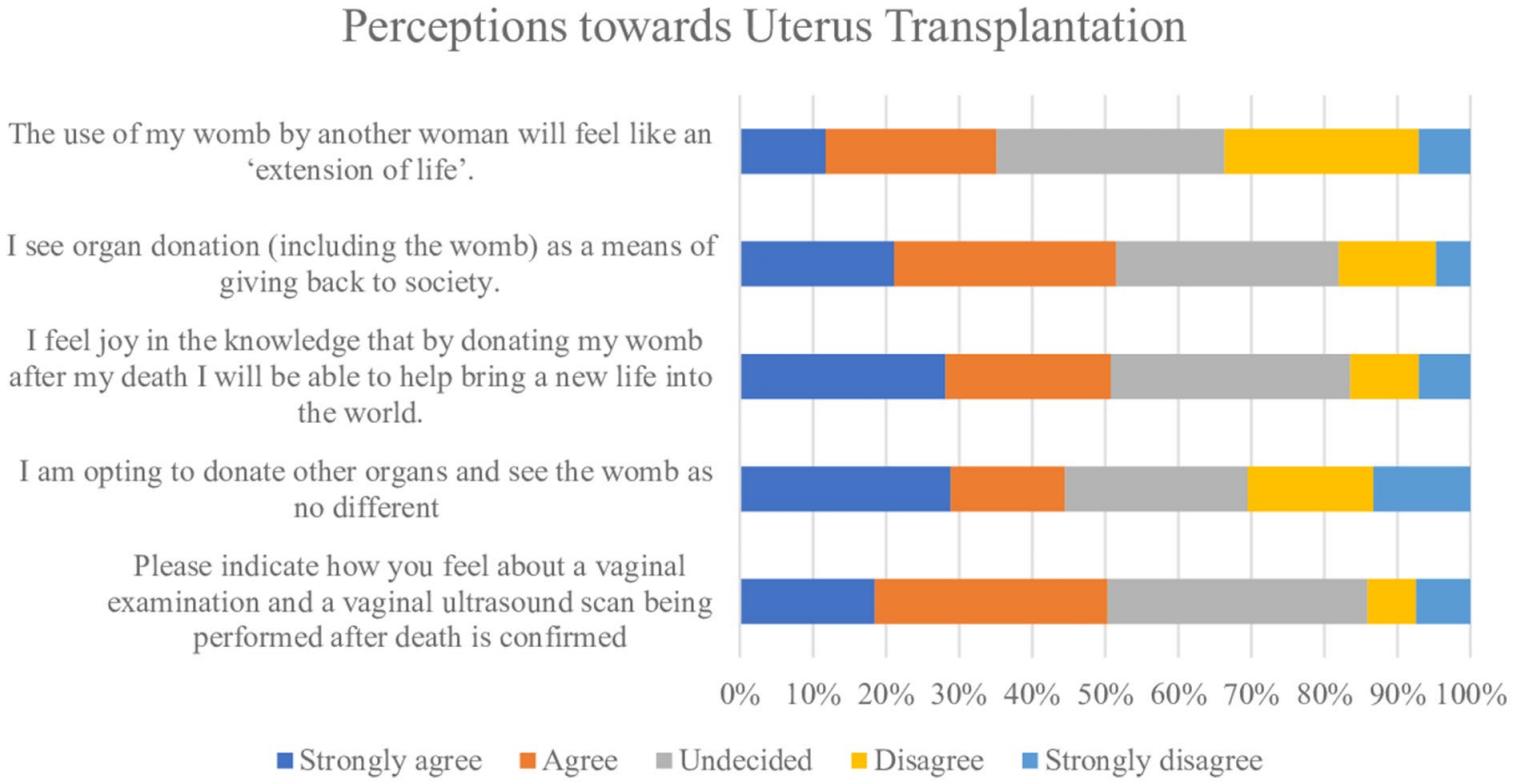
Perceptions toward uterus transplantation.

52% of women (*n* = 74) wished for their family to be informed of the outcome of the uterus donation such as a birth if they had donated. More than half (*n* = 68, 51%) strongly agreed or agreed to a vaginal examination and a vaginal ultrasound being performed, following confirmation of death on the potential uterus donor and only 7% (*n* = 10) strongly disagreed (Figure 5). The majority of women (*n* = 80, 56%) were not concerned about attention from the media if they were to donate their uterus.

**Figure 6:**
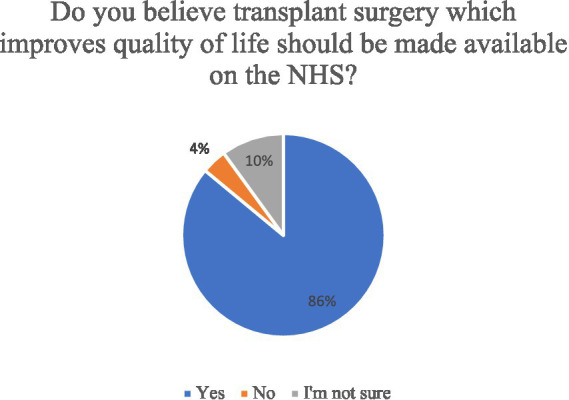
Opinion on public funding quality of life improving transplants.

Potential barriers to uterus donation were explored (Figure 3). Twenty-nine women (23%) strongly agreed or agreed they were not intending to donate their uterus as it was not a life-saving transplant. Most women (*n* = 123, 86%) strongly agreed or agreed on believing that quality of life improving transplants should be made available on the NHS (Figure 6) and this figure was higher (*n* = 51, 98%) among the group who responded they would not consider to donate their uterus after death (*n* = 77, 54%). Participants were asked if they think the birth of a child from a transplanted uterus has genetic links to the deceased womb donor, and overall, 29% (*n* = 42) women said yes. There was little variation among the age groups on this belief with 52% of women less than 30 years (*n* = 22) and 48% of women ≥30 years *n* = 20, responding with yes.

Interestingly, among the women who were not considering to donate their uterus, 78% (*n* = 38) thought the child from a transplanted uterus had genetic links to the deceased donor.

On the possibility of a transgender male to female woman being a uterus transplant recipient in the future, 30% (*n* = 39) strongly agreed or agreed they would be happy to donate their uterus to help them achieve a pregnancy.

## Discussion

### Main findings

This is the first study reporting on the attitudes, knowledge and perceptions among the general public toward UTx and uterus donation in the United Kingdom. The findings indicate positive attitudes and perceptions toward uterus donation but a low level of awareness and knowledge of UTx in general.

The responses indicate the majority of women were unaware the uterus was not included on the opt out organ donation register and required separate consent. Age appeared influential in this, with those ≥30 years displaying higher awareness rates compared to the cohort below age 30 years. Most women in this study intending to donate their organs also intend to donate their uterus. Atheists were most likely to consider donating their uterus after death followed by women of the Muslim and Christian faith. Participants of a White ethnicity were also more likely to consider uterus donation compared to other ethnic groups.

Among the majority who were intending to donate their organs, the motivational themes included giving back to society and believing organs are precious commodities which are best put to use after death. This concern for the wellbeing of others is consistent with a previous study on women willing to become living UTx donors where the main motivational factor was to help other women carry and give birth to their own children (22). Similarly, among living kidney donors, one of the main influencing factors for donation was found to be compelling feelings of altruism and inherent responsibility (23). Interestingly, the younger cohort (age less than 30 years) were more likely to opt out of organ donation than those ≥30 years. This may be indicative of a lack of education around donation and perhaps fears of the organ procurement process. Studies have shown younger cohorts are less likely to engage in organ donation where registration is concerned, likely to be due to them thinking less seriously about their mortality (24). However, this is the first study highlighting age being a factor influencing “opting out” of organ donation, and is a poignant factor which needs to be explored further.

We found a significant proportion of participants wanted to donate their uterus, viewing it as an extension of life and a gesture which would bring joy through new life. The majority of respondents were childless but intended to have children. However, the intention to have a child did not result in them being more likely to donate their uterus after death, but rather personally knowing somebody experiencing infertility. Interestingly, the majority of participants who were not willing to donate their uterus after death felt the child born following a UTx would carry genetic links to the uterus donor. Additionally, there was a very similar distribution among the age groups (< 30 years and ≥ 30 years) with regards to concerns around a genetic link to the uterus donor. This is an important element to consider in future educational campaigns.

With regards to uterine donation, most women were not concerned about an additional vaginal examination being performed as part of the preoperative assessment, and were not concerned about the additional media attention they or their families may receive.

### Strengths and limitations

This study has a number of advantages including a strong response rate, a representative population of potential uterus donors and the first study of a population-based ascertainment of views toward uterus donation. Other advantages include a unique insight into views toward organ donation in general and intentions for donating. Its limitations include a lack of family centered data which would help to establish the views of family members of uterus donors. This would provide a useful insight on the potential barriers raised by families preventing them from authorizing uterus donation after death.

### Interpretation (in light of other evidence)

The participants were well distributed in their age profile which is representative of the potential United Kingdom deceased donor cohort available for the INSITU study.

Interestingly, although two thirds of women reported knowing nothing at all on UTx, 43% answered they would consider donating their uterus. This represents a considerable potential for the once predicted limited pool of uteri donors (19). Though, greater awareness of the aims of the procedure and the potential recipient cohort will likely improve upon this rate. In a survey regarding face transplants, willingness to donate improved and negative decisions were reversed with increased awareness of the disfigurements of patients in need and through shared knowledge of the number of procedures performed worldwide (25).

The religious and sociocultural background of the respondents may have influenced the number willing to donate their uterus as demonstrated in the findings of Atheist and those of a White ethnicity being the largest cohort willing to donate. The most prevalent faith group was Islam followed by Christianity and over half of all respondents were of an Asian background. Although this can be said to be representative of the diverse population of the United Kingdom, studies have demonstrated a low rate of donation among Black Asian and Minority Ethnic (BAME) and faith groups (26). This is perhaps the most important cohort for future education campaigns around uterus donation. The United Kingdom Potential Donor Audit showed for eligible DBD donors there was a significant difference (*p* < 0.0001) between the consent/authorization rates among the families of white (81%) and BAME donors (35%) (27). Additionally, contrary to the opinion held by donors on religious beliefs, the majority opinion among faith leaders in the United Kingdom is that organ donation is infact permitted (28). However, this knowledge may not have filtered into the public domain- one reason for this being the low prioritization of organ donation discussions among faith groups (28). The willingness for uterus donation may be further enhanced with organized dialog among these groups.

There was a strong consensus among respondents on feeling joy by helping to bring a new life into the world through uterus donation. This is similar to the DUETs study on a donor pool in the United States and the United Kingdom based study on potential living uterus donors who reported their motivation to donate being a desire to enable a woman to carry a child of her own. Thus this is perhaps a poignant reflection on the value women place on the gestation of one’s biological child (22, 29).

Among women who were concerned the child born in a transplanted uterus carried genetic links to the deceased womb donor, the majority responded they would not consider donating their uterus after death. This provides a novel insight into the concerns and misconceptions held by potential uterus donors and offers a key point for which to base future information campaigns.

Most women were keen for their family to be informed of the outcome of the donation. This is interesting as it suggests potential uterus donors do wish for their families to share the joy of childbirth. Studies focused on donor family experiences in the post donation phase reveal the decision to donate does not lessen the burden of loss and suffering experienced due to the loss of a loved one and in some cases feelings of regret are prevalent following their decision to agree to the donation (30–32). Thus it is likely the news of a positive outcome post donation will better manage the needs of the donor family at such a sensitive time.

Less than a quarter of women in this study disagreed on donating their uterus based on the notion that it is not a life-saving transplant. Discussions around the value of a UTx in the context of other life-saving transplants continue to be published and are beyond the remit of this study. Given the proportion of women who did not disagree, it is evident the value of uterus donation among potential organ donor’s rests not just on saving a life but rather on the concept of enhancing another woman’s life. Most participants also believed quality of life improving transplants should be made available on the NHS thus indicating a positive attitude toward the public provision of UTx.

One of the other key factors which would influence the success of a UTx clinical program is the attitudes of the involved healthcare personnel. A previous study by our team indicated excellent overall support from clinical staff with 94% of those surveyed in favor of UTx (33).

This study provides an insight into the public’s attitude toward UTx which will be the most determinant factor overall. The findings in this study are suggestive of the need for a wider, national conversation on UTx and what donation would entail.

## Conclusion

This study presents the views of the members of the public toward UTx and uterus donation. Findings indicate a favorable opinion toward UTx and a positive attitude toward donation of the uterus. The study highlights the need for greater public education around UTx. Themes such as the utility of UTx, the potential recipient profile and the absence of any genetic link to the resultant child need addressing. Women interested in donating their uterus need to be encouraged to share their donation decision with family and friends in order to help improve the family consent and authorization rate when uterus donation is in question.

## Data availability statement

The original contributions presented in the study are included in the article/Supplementary material, further inquiries can be directed to the corresponding author.

## Ethics statement

The studies involving human participants were reviewed and approved by Imperial College London. The patients/participants provided their written informed consent to participate in this study.

## Author contributions

SV conceived the study, designed and developed the questionnaire, and performed data analysis. SSh helped in recruiting eligible participants. BJ, SSa, IQ, and JS helped in the preparation and review of the manuscript. All authors contributed to the article and approved the submitted version.

## Conflict of interest

The authors declare that the research was conducted in the absence of any commercial or financial relationships that could be construed as a potential conflict of interest.

## Publisher’s note

All claims expressed in this article are solely those of the authors and do not necessarily represent those of their affiliated organizations, or those of the publisher, the editors and the reviewers. Any product that may be evaluated in this article, or claim that may be made by its manufacturer, is not guaranteed or endorsed by the publisher.
