# Supplementary material for: Attitudes, knowledge, and perceptions among women toward uterus transplantation and donation in the United Kingdom
Source: Front Med (Lausanne). 2023 Aug 16;10:1223228. doi: 10.3389/fmed.2023.1223228 (PMC10467283; doi:10.3389/fmed.2023.1223228)
Supplement: Supplementary file 1 [file Data_Sheet_1.PDF]

Imperial College London

Research Study: Participant Information Sheet

Public perceptions towards womb transplantation

Principal Investigator: Mr J Richard Smith
Co-Investigators: Dr Saaliha Vali, Mr Benjamin Jones

We invite you to take part in this research

Before you decide, it is important for you to understand why the research is being done and what it will involve. Please take time to read the following information carefully and discuss it with others if you wish.

Ask us if there is anything that is not clear or if you would like more information. Take time to decide whether or not you wish to take part.

Thank you for reading this.

Background

A small number of women worldwide suffer with infertility secondary due to the absence of a womb or a disease rendering the womb unable to carry a pregnancy. Previously, for these women the only option was surrogacy or adoption. However, in the UK we are running a clinical trial on the transplantation of the womb, which allows for these women to carry their own child following IVF.

The current options to acquire motherhood for women without a functional womb include adoption or surrogacy. However, not only are these options associated with complex legal, financial, cultural, ethical and religious factors, but they do not offer the women the opportunity to experience pregnancy. Womb transplantation provides an opportunity to overcome these issues whilst also allowing these women to conceive and carry a pregnancy themselves with the help of IVF.

More than 70 procedures have now been performed worldwide, and at least 24 babies have been born as a result, thus proving womb transplantation as a viable option.

What are the benefits?

You will benefit from learning a little more about womb transplantation. Also, by taking part you have helped to take the research a step further and in time will have helped a number of women with absolute uterine factor infertility to achieve motherhood.

© Imperial College of Science, Technology and Medicine

Imperial College London

What is the purpose of the study?

As this is a procedure which is new for the U.K, little is known on the public perception and acceptability. This study aims to explore the public opinion on womb transplantation and the donation of the womb after death. We hope that in improving our understanding of the public perception of this we can help to navigate solutions to increase awareness and willingness to donate.

Why have I been invited?

You have been invited to partake in this study as part of an effort to understand the public perception towards womb transplantation and donation. This questionnaire has been advertised on social media platforms to all qualifying members of the public. We are aiming to have approximately 100-300 responses.

Do I have to take part?

It is up to you to decide whether or not to take part. If you do decide to take part you will be given this information sheet to keep and be asked to sign a consent form. If you decide to take part you are still free to withdraw at any time and without giving a reason.

What will happen to me if I take part?

The only thing required of you is to read this information sheet, give consent and complete the questionnaire. There will be no follow up once you have submitted the questionnaire.

What do I have to do?

After reading this participant information leaflet, which explains the conduct of the study, you will give consent and complete the questionnaire, using the link provided. It should take approximately 10 minutes to complete. Following the completion of the questionnaire, no further action will be required.

What are the possible disadvantages and risks of taking part?

There are no disadvantages or risks to your partaking in this questionnaire, other than the time taken to complete it.

What are the possible benefits of taking part?

This study will give you the opportunity to express your views on womb transplantation and donation. Whilst the results may not directly influence you, your responses, along with the other responses

© Imperial College of Science, Technology and Medicine

received, will determine the need for our research team to engage with the public on improving the awareness and understanding of womb transplantation and donation.

What will happen to the results of the research study?

The results from this study will be used directly by the womb transplant research team to better understand the public perceptions. The data collected will also be used for publication in peer reviewed journals and included in oral and poster presentations at national and international conferences. You will not be able to be identified in any publications or presentations.

Who is organising and funding the study?

Imperial College London is the study sponsor. This study has been organised by the registered charity Womb Transplant UK (Charity no 1138559). No healthcare professional will receive payment for conducting this research.

Who has reviewed the study?

This study was given approval by Head of Department and Research Governance Integrity Team (RGIT).

Contact for further information

If you have any further questions please do not hesitate to contact the study coordinator, Dr Saaliha Vali, at info@wombtransplantuk.org who will be able to answer any queries

Thank you for reading this leaflet and considering whether to take part in the study

© Imperial College of Science, Technology and Medicine
