# Supplementary material for: Attitudes, knowledge, and perceptions among women toward uterus transplantation and donation in the United Kingdom
Source: Front Med (Lausanne). 2023 Aug 16;10:1223228. doi: 10.3389/fmed.2023.1223228 (PMC10467283; doi:10.3389/fmed.2023.1223228)
Supplement: Supplementary file 2 [file Data_Sheet_2.PDF]

Online Questionnaire (V1 - 4/01/2021)

Full title: Public perceptions towards womb transplantation

This study aims to gain a greater insight into the perceptions and motivations of members of the public towards womb transplantation. This is a relatively new option for women who have absolute uterine factor infertility, where they have either been born without a womb or had the womb removed for medical reasons. The procedure involves a donated womb from a living or a deceased donor. Following the transplant, IVF and embryo transfer allows for the woman to carry her own child.

As this is a procedure which is new for the U.K, little is known on the public perception and acceptability. This study aims to explore the public opinion on womb transplantation and the donation of the womb after death. We hope that in improving our understanding this we can help to navigate solutions to increase awareness and willingness to donate.

Please complete the following questionnaire after reading the participant information sheet (V1 19-04-21) and providing consent.

I confirm that I have read and understand the participant information sheet v. 1 19.04.21 for the above study

I understand that my participation is voluntary, and I am free to withdraw at any time before submission of the questionnaire, without giving any reason and without my legal rights being affected.

I confirm I am over 16 years of age.

I consent to take part in the study

QUESTIONNAIRE

Section 1: Demographic information

a) Age (years)

16-19 ☐
20-29 ☐
30-39 ☐
40-49 ☐
50-59 ☐
60+ ☐
Would rather not say ☐

b) Ethnicity

White ☐
Asian ☐
Black ☐
Mixed ☐
Other ☐
Would rather not say ☐

c) Employment status

Employed (Full time) ☐
Employed (Part time) ☐
Self Employed ☐
Student ☐
Housewife ☐
Unemployed ☐
Would rather not say ☐

d) Religion

Christian ☐
Muslim ☐
Hindu ☐
Other ☐
Athiest ☐
Would rather not say ☐

e) Relationship status

Single ☐
Living with partner ☐
Married ☐
Divorced ☐
Separated ☐
Widowed ☐
Would rather not say ☐

f) **Educational attainment:** please select the highest level of qualification you hold:

No formal qualification ☐

Level 1 (1-4 GCSEs, Scottish Standard Grade or equivalent qualifications). ☐

Level 2 (5 + GCSEs, Scottish Higher, Scottish Advanced Higher or equivalent qualifications). ☐

Apprenticeship (Apprenticeships (England, Wales and Northern Ireland only). ☐

Level 3 (2 + A-levels, , HNC, HND, SVQ level 4 or equivalent qualifications). ☐

Level 4 or above (First or higher degree, professional qualifications or other equivalent higher education qualifications). ☐

Other qualifications (Other vocational / work related qualifications and non-UK / foreign qualifications (England, Wales and Northern Ireland only). ☐

g) **Medical history: please select all that apply to you**

History of heart/lung/other disease (please state). Yes ☐ No ☐

……………………………….

History of cancer Yes ☐ No ☐

History of high blood pressure in pregnancy Yes ☐ No ☐

More than 2 caesarean sections Yes ☐ No ☐

Any children born 'preterm' or at <37weeks Yes ☐ No ☐

Surgery to the womb (please state) Yes ☐ No ☐

……………………………………..

Miscarriage (please state number) Yes ☐ No ☐

……………………………………..

Surgery to the cervix Yes ☐ No ☐

Section 2: Knowledge and awareness of UTx

1. **Are you aware of the new 'opt out' system for organ donation?**
   Yes ☐ No ☐

2. **Will you be opting out of organ donation?**
   Yes ☐
   No ☐
   I wish to donate my organs ☐

3. **Are you aware donation of the womb is not included on the organ donor register?**
   Yes ☐ No ☐

4. **Do you know of anyone who:**

   Has received an organ (any) Yes ☐ No ☐

   Had donated an organ (any) Yes ☐ No ☐

5. **Do you have children?** Yes ☐ No ☐

If Yes how many ☐

If No:

I do not intend to have children ☐

I have experienced infertility ☐

I intend to have children in the future ☐

6. **Do you know of anybody personally who is experiencing infertility?**

Yes ☐ No ☐

If Yes (please select one of the options below)

Sibling ☐

Relative ☐

Friend ☐

Colleague ☐

7. **Have you previously heard of the concept of womb transplantation?**

Yes ☐ No ☐

If Yes:

Through the Media ☐

Friend ☐

I have researched it for myself ☐

8. **Were you aware of the option of donating your womb after death?**

Yes ☐

No ☐

9. **How much do you know about womb transplantation?**

A lot ☐ A fair amount ☐ Heard it discussed only a few times ☐ Nothing ☐

10. **I understand the benefits of womb transplantation.**

Strongly agree ☐ Agree ☐ Undecided ☐ Disagree ☐ Strongly Disagree ☐

If disagree/strongly disagree, why? ………..………………………………

11. **Do you think the birth of a child from a transplanted womb has genetic links to the deceased womb donor?**

Yes ☐

No ☐

12. **If you were to donate your womb would you wish for your family to be informed of the outcome following donation such as the birth of a child?**

Yes ☐

No ☐

13. **Do you believe in transplant surgery which improves the quality of life should be made available on the NHS? (Examples of this include hand and retina transplants).**

Yes ☐

No ☐

I'm not sure ☐

14. **Given womb transplants are a new procedure in the U.K, are you concerned about attention from the media and intrusion into your personal life if you donate your womb?**

Strongly agree ☐ Agree ☐ Undecided ☐ Disagree ☐ Strongly Disagree ☐

If disagree/strongly disagree, why? ………..…………………………………………

15. **After death is confirmed, if donating the womb, a vaginal examination (with a speculum) and a vaginal ultrasound scan is performed by the Gynaecology team to assess if the womb is suitable. Please indicate how you feel about this.**

Strongly agree ☐ Agree ☐ Undecided ☐ Disagree ☐ Strongly Disagree ☐

If disagree/strongly disagree, why? ………..…………………………………………

16. **Would you consider donating your womb after death?**

Yes ☐
No ☐
N/A as I do not have a uterus ☐

Section 3: Perceptions towards UTx

Please indicate if you agree/disagree with the following statements:

17. I am opting to donate other organs and see the womb as no different

Strongly agree ☐ Agree ☐ Undecided ☐ Disagree ☐ Strongly Disagree ☐

If disagree/strongly disagree, why? ……….…………………………………………

18. I feel joy in the knowledge that by donating my womb after my death I will be able to help bring a new life into the world.

Strongly agree ☐ Agree ☐ Undecided ☐ Disagree ☐ Strongly Disagree ☐

If disagree/strongly disagree, why? ……….…………………………………………

19. I see organ donation (including the womb) as a means of giving back to society.

Strongly agree ☐ Agree ☐ Undecided ☐ Disagree ☐ Strongly Disagree ☐

If disagree/strongly disagree, why? ……….…………………………………………

20. All organs including the womb are precious resources which are best put to use after death.

Strongly agree ☐ Agree ☐ Undecided ☐ Disagree ☐ Strongly Disagree ☐

If disagree/strongly disagree, why? ……….…………………………………………

21. The use of my womb by another woman will feel like an 'extension of life'.

Strongly agree ☐ Agree ☐ Undecided ☐ Disagree ☐ Strongly Disagree ☐

If disagree/strongly disagree, why? ……….…………………………………………

22. I personally don't agree with organ donation in general.

Strongly agree ☐ Agree ☐ Undecided ☐ Disagree ☐ Strongly Disagree ☐

If disagree/strongly disagree, why? ……….…………………………………………

23. I personally don't agree with donation of the womb.

Strongly agree ☐ Agree ☐ Undecided ☐ Disagree ☐ Strongly Disagree ☐

If disagree/strongly disagree, why? ……….…………………………………………

24. I do not intend to donate my womb as it's not a lifesaving transplant.

Strongly agree ☐ Agree ☐ Undecided ☐ Disagree ☐ Strongly Disagree ☐

If disagree/strongly disagree, why? ……….…………………………………………

25. Religious barriers prevent me from donating my womb.

Strongly agree ☐ Agree ☐ Undecided ☐ Disagree ☐ Strongly Disagree ☐

If disagree/strongly disagree, why? ……….…………………………………………

26. Culturally, donation of my womb after death is unacceptable.

Strongly agree ☐ Agree ☐ Undecided ☐ Disagree ☐ Strongly Disagree ☐

If disagree/strongly disagree, why? ……….…………………………………………

27. I am worried about a fast tracked brain death diagnosis if I sign up to be an organ donor.

Strongly agree ☐ Agree ☐ Undecided ☐ Disagree ☐ Strongly Disagree ☐

If disagree/strongly disagree, why? ……….…………………………………………

28. I am concerned about not 'being whole' after death if I was to donate.

Strongly agree ☐ Agree ☐ Undecided ☐ Disagree ☐ Strongly Disagree ☐

If disagree/strongly disagree, why? ………..…………………………………………

29. I do not wish to donate my organs as I have a fear of surgical procedures.

Strongly agree ☐ Agree ☐ Undecided ☐ Disagree ☐ Strongly Disagree ☐

If disagree/strongly disagree, why? ………..…………………………………………

30. My family disagree with organ donation which has affected my decision not to donate.

Strongly agree ☐ Agree ☐ Undecided ☐ Disagree ☐ Strongly Disagree ☐

If disagree/strongly disagree, why? ………..…………………………………………

31. I wish to donate my organs but not my womb (please specify why).

Strongly agree ☐ Agree ☐ Undecided ☐ Disagree ☐ Strongly Disagree ☐

If disagree/strongly disagree, why? ………..…………………………………………

32. In the future womb transplantation may be possible for transgender women.

Would you be happy for your womb to be donated to a transgender woman in order for them to achieve a pregnancy and have children??

Strongly agree ☐ Agree ☐ Undecided ☐ Disagree ☐ Strongly Disagree ☐

If disagree/strongly disagree, why? ………..…………………………………………

END
